# Dataset for WWW landing pages webobject retrieval performance evaluation

**DOI:** 10.1016/j.dib.2020.105429

**Published:** 2020-03-14

**Authors:** Patrick Seeling

**Affiliations:** Department of Computer Science, Central Michigan University, United States

**Keywords:** Performance evaluation, World Wide Web, Web caching, Information retrieval

## Abstract

This dataset describes data obtained from a multi-day World Wide Web (WWW) measurement campaign distributed internationally across multiple Amazon Web Service (AWS) datacentres. The Chrome web browser was controlled by the Selenium framework to make repetitive requests to several popular websites; the resulting webobjects were captured by a proxy server and details about them stored in the provided SQLite3 databases. A Python script is provided to evaluate the webobjects with respect to their configured as well as their actual expiration times, as part of our more detailed analysis that we provide in [1]. Researchers and practitioners can readily employ this dataset in their own research endeavours with little efforts for avenues of inquiry beyond webobject expiration times we described in [1], as we provide additional information about each webobject and each website visit during the measurement campaign time horizon.

Specifications table

**Table:** 

Subject	Computer Networks and Communications
Specific subject area	World Wide Web and content delivery performance evaluation
Type of data	Databases Tables
How data were acquired	Virtual machines at various Amazon Web Services (AWS) locations were employed to execute a Selenium-controlled Chrome web browser without caching. A local proxy server was used in the middle to capture requests and determine hashes (SHA-1) of exchanged webobjects.
Data format	Filtered Analysed
Parameters for data collection	Web landing pages for several popular websites were chosen. Selection of websites includes several that can be approximately categorized similarly, e.g., Google and Bing as search websites.
Description of data collection	The data was collected over multiple days through repeated scripted browser visits to a selection of web landing pages without browser caching. A custom intermediate proxy server was employed to intercept the browser requests for webobjects and they were logged together with a unique identifier (SHA-1 checksum). The logs were subsequently parsed into databases, which were subsequently utilized to generate contingency-table data for requests.
Data source location	Data sources were virtual machines hosted in Amazon Web Services (AWS) datacentres at multiple worldwide locations: Frankfurt (Germany), Oregon (USA), Sao Paulo (Brazil), Sydney (Australia), Tokyo (Japan), and Virginia (USA).
Data accessibility	Deposited for all locations as recommended into Mendeley Data. We have included a single data set for *Virginia* with this submission for convenience. Repository name: Mendeley Data Data identification number: Direct URL to data: http://dx.doi.org/10.17632/3rkwskk2fn.1
Related research article	Patrick Seeling WWW Retrieval Handling Optimization $w_{\rho }^{3}$: A Metric for Webpage Timeout Setting Performance Evaluation and Comparison Future Generation Computer Systems

## Value of the data

• Shared longer-term data from worldwide website access to conduct research into the complexity and delivery intricacies of modern web pages will aid in the understanding of the dynamics of these pages over time [2].
• Researchers, engineers, and industry experts will find the provided data article useful to develop ground-truth approaches for web delivery optimization models and simulations [3].
• Statistical analyses and other evaluations can be performed based on the available data, including evaluations of global differences due to worldwide data source locations.
Web landing pages are the first items a user experiences but feature significant numbers of webobjects and data amounts, and optimization approaches can greatly benefit users and service providers.

## 1. Data

We provide two types of data with this Data in Brief article, namely (i) compressed (ZIP format) SQLite3 databases and (ii) comma-separated value (CSV) files. The databases contain detailed information about the webobjects that constitute the browser-based request-responses to display one of multiple popular websites’ landing webpages (e.g., http://www.amazon.com), as further detailed in Table 1.

**Table 1:** Content overview of the SQLite3 databases for this article, including data types and brief descriptions.

Column name	Data type	Description
reqID	INTEGER (key)	General row index for the table
requestTime	INTEGER	The time of the original request (epoch in ms)
sha1	TEXT	The SHA-1 checksum of the delivered webobject
url	TEXT	The URL requested
expires	TEXT	The set or determined expiration time (epoch in ms)
contentLength	INTEGER	The size of the webobject in Bytes
expirationMissing	INTEGER	Flag indicating if the expiration was set due to no server hints
siteVisited	TEXT	Website visited
contentType	TEXT	The content-type HTTP header entry of the webobject
responseTime	INTEGER	Time the response was received (epoch in ms)

These databases were employed to generate the statistics that can be found in the individual CSV files for each of the websites visited and for which data is present in the databases. The CSV files contain the data described in Table 2. We note that some of these values are modified during their generation (we treat missing expirations as immediate expirations and determine modified versions of the TN/FN in determining the MCC and DMCC) as described in greater detail in the accompanying paper [1] and shown in the source code sample below.

**Table 2:** Columns in the individual website CSV files and their brief description.

Column name	Description
DFN	Data-based contingency table False Negatives (in kByte)
DFP	Data-based contingency table False Positives (in kByte)
DMCC	Data-based Matthew's Correlation Coefficient
DTN	Data-based contingency table True Negatives (in kByte)
DTP	Data-based contingency table True Positives (in kByte)
FN	Webobject count-based contingency table False Negatives
FP	Webobject count -based contingency table False Positives
MCC	Webobject count-based Matthew's Correlation Coefficient
TN	Webobject count-based contingency table True Negatives
TP	Webobject count-based contingency table True Positives
Revisit	Time between the original and next visit of the website, for the following discrete re-visit scenarios (in seconds): 0, 1000, 15,000, 30,000, 60,000, 300,000, 900,000, 1,800,000, 3,600,000, 21,600,000, 43,200,000, 86,400,000
Site	Website label (reading-friendly from Table 1) for the following URLs: http://www.Linkedin.com, http://www.Twitter.com, http://www.Tumblr.com, http://www.google.com, http://www.Chase.com, http://www.Wikipedia.org, http://www.amazon.com, http://www.Imgur.com, http://www.Reddit.com, http://www.Ebay.com, http://www.Craigslist.org, http://www.Yahoo.com, http://www.Netflix.com, http://www.facebook.com, http://www.Cnn.com, http://www.Pinterest.com, http://www.Live.com, http://www.Youtube.com, http://www.Bing.com, http://www.Go.com
Start	Timestamp of the original website visit (from Table 1), e.g., ranging from 1,461,941,849,142–1,462,564,798,155 for one site (corresponding to Friday, April 29, 2016 14:57:29.142 through Friday, May 6, 2016 19:59:58.155) and slightly different times for others.

Data is organized into different folders for Amazon Web Services (AWS) datacentre source locations worldwide, namely Frankfurt (Germany), Oregon (USA), Sydney (Australia), Sao Paulo (Brazil), Tokyo (Japan), and Virginia (USA).

## 2 Experimental design, materials, and methods

Specifically, the dataset presented here is motivated by our initial works presenting a comparison of fixed and mobile website landing pages in [2], which also performs an investigation of the webobject types and their impacts on a webpage's size over time. More recently, our evaluations in [4] focused on webpage interactions, similar to the presented content here, but with a focus on webobjects delivered via HTTP and HTTPS and the potential negative impacts on already established content caching schemes. Our dataset contains the same information used for these prior works, but from a slightly different time period matching the one employed in [1]. The dataset and its contained information can readily be used in the context of each of these prior works’ viewpoints to evaluate schemes to address the challenges described in these prior works. The dataset generation is described in the overall setup sections of these prior works and briefly discussed here to make this contribution self-contained and readily usable for researchers and practitioners.

We generated a virtual machine environment in AWS that combined the Windows operating system, a modified local proxy server, Chrome browser, and Selenium browser control framework. The virtual machine was deployed at several AWS datacentres to generate the source data as follows. Each website's landing webpage described in the dataset was sent to the browser, which had its local caching disabled. The browser's requests for webobjects were logged by the proxy server and the responses were matched to the requests. After receipt, the proxy server determined the SHA-1 checksum for the webobject and logged the data while forwarding the webobject to the requesting browser. Both browser and proxy server were additionally configured to also request HTTPS (i.e., securely) delivered webobjects. The overall scripting approach went through the range of websites and subsequently paused for about 10–15 min before repeating the requests. Slight timing deviations are normal here, as intermediate webobject requests could require more time to be fulfilled. The resulting logfiles were parsed into the databases described in Table 1.

The databases were employed to generate the contingency tables for webobject counts and sizes further described in [1] through scripted parsing with Python. We note that missing expirations are treated as immediate expirations overall, as a browser or cache would need to resume to the common webobject retrieval from the source if no caching hints are available. In the following, we provide an abbreviated pseudocode for the main logic that produces the CSV file content for each start time of a Site's visit with different Revisit times (as in Table 2) as:

FOREACH **Revisit** time:

  FOREACH link in **Site**:

    IF set expiration time <= **Revisit** THEN

       EXP(link) = TRUE

    ELSE

       EXP(link) = FALSE

    IF time of checksum change <= **Revisit** THEN

       MOD(link) = FALSE

    ELSE

       MOD(link) = TRUE

  SET TP, TN, FP, FN = 0

  FOREACH link in **Site**:

    IF EXP(link) AND MOD(link) THEN

       INCREMENT TP

    IF !EXP(link) AND !MOD(link) THEN

       INCREMENT TN

    IF EXP(link) AND !MOD(link) THEN

       INCREMENT FP

    IF !EXP(link) AND MOD(link) THEN

       INCREMENT FN

  IF (FN == 0) AND (FP > 0) THEN

    INCREMENT FN

  IF (TN == 0) AND (TP > 0) THEN

    INCREMENT TN

  SET MCC = TP * TN - FP * FN

  SET bot = squareroot(TP+FP) * squareroot(TP+FN) * squareroot(TN+FP) * squareroot(TN+FN)

  IF bot != 0 THEN

    SET MCC = MCC / bot

We initially note that the outlined pseudocode handles the *counts* of the webobjects, with the data-centric versions readily obtainable through multiplication of the respective individual webobject with its size. We furthermore note that the MCC and DMCC values generated are slightly different from their standard definitions, with reasoning outlined in [1]. Modifying and implementing the provided pseudo-code to produce variations for other research and implementation efforts should be straightforward.

## Appendix Supplementary materials

Image, application 1

Image, application 2
